# ArtiLock: Smartphone User Identification Based on Physiological and Behavioral Features of Monosyllable Articulation

**DOI:** 10.3390/s23031667

**Published:** 2023-02-02

**Authors:** Aslan B. Wong, Ziqi Huang, Xia Chen, Kaishun Wu

**Affiliations:** 1 College of Computer Science and Software Engineering, Shenzhen University, Shenzhen 518061, China; 2 Department of Psychology, University of Wisconsin-Milwaukee, Milwaukee, WI 53211, USA; 3 Information Hub, The Hong Kong University of Science and Technology (Guangzhou), Guangzhou 511453, China

**Keywords:** biometric, articulation sensing, human–computer interactions, ubiquitous computing, mobile interfaces, smart sensing

## Abstract

Although voice authentication is generally secure, voiceprint-based authentication methods have the drawback of being affected by environmental noise, long passphrases, and large registered samples. Therefore, we present a breakthrough idea for smartphone user authentication by analyzing articulation and integrating the physiology and behavior of the vocal tract, tongue position, and lip movement to expose the uniqueness of individuals while making utterances. The key idea is to leverage the smartphone speaker and microphone to simultaneously transmit and receive speech and ultrasonic signals, construct identity-related features, and determine whether a single utterance is a legitimate user or an attacker. Physiological authentication methods prevent other users from copying or reproducing passwords. Compared to other types of behavioral authentication, the system is more accurately able to recognize the user’s identity and adapt accordingly to environmental variations. The proposed system requires a smaller number of samples because single utterances are utilized, resulting in a user-friendly system that resists mimicry attacks with an average accuracy of 99% and an equal error rate of 0.5% under the three different surroundings.

## 1. Introduction

In today’s information society, mobile devices store data related to personal privacy, such as account information stored in electronic banks and private chats in social networking software, which makes user authentication on these devices crucial. Generic authentication methods require users to enter text passwords or gestures consisting of letters, numbers, symbols, and so on. In terms of mobile authentication, where passwords are increasingly common, voice authentication is becoming increasingly attractive as an alternative to passwords, and the market is expected to grow by 22.8% per year from USD 1.1 billion in 2020 to USD 3.9 billion by 2026 [1]. There is high demand for voice authentication in mobile systems. For instance, Google integrates voice authentication into its Android operating system to allow users to authenticate their voices [2]. Voice authentication is a secure method of logging into mobile apps and devices to access financial services through mobile devices such as WeChat [3], HSBC [4], and Citi [5].

Biometric user authentication methods are based on human physiological characteristics, including the iris, voice print, fingerprints, and face. Fingerprint-based unlocking schemes are among the most widely used authentication methods, in which fingerprints are collected through fingerprint sensors embedded in the device. Although each person’s fingerprint is different, this feature can be replicated precisely by specific means to successfully deceive an authentication system [6]. Moreover, if the finger is covered with water or other liquids, the effectiveness of the sensor is significantly reduced. Face-based unlocking methods, such as Apple’s face ID [7], have become popular in recent years. However, this method is sensitive to the light intensity of the surrounding environment and places more requirements on a user’s face. For example, the face must be free from blatant obstructions.

Although voice authentication is generally secure, the voiceprint-based authentication method has the drawback of being affected by environmental noise, long passphrases, and large numbers of registered samples. More user-friendly authentication techniques have been proposed based on automatic speech recognition (ASR) to overcome the shortcomings of traditional voice authentication approaches based on speech signals, such as physiological and behavioral speech characteristics. Consequently, the approaches mentioned above rely on multiple syllables to produce unique features. However, these two approaches still require more than one syllable for authentication passphrases. In summary, two main challenges arise with this type of research: (1) the registration stage requires large samples, and (2) it is susceptible to the dynamic nature of environmental noise interference.

The rapid development of smartphones has triggered research on motion recognition to realize user authentication through speech articulation. Our work aims to employ individual physiological speech characteristics and behavioral characteristics of the vocal tract, tongue, and lip movements to develop a novel method for smartphone user authentication. Because Doppler effect analysis is typically used in pattern recognition with ultrasonic signals, we face several challenges in practice. First, because speaking habits involve both static shape and dynamic movements of the lip and tongue, we should accurately characterize the articulation or speech organs when speaking with speech and ultrasonic signals. Second, because the ultrasonic signals generated by mobile devices can easily interfere with physically insecure spaces, filters should be designed to resist noise. There has been no study on the articulation of single utterances for articulation-based user identification or authentication; therefore, the third challenge is that the proposed system requires less calculation in terms of the two aforementioned challenges, which are resistant to attack and environmental noise.

**Figure 1:**
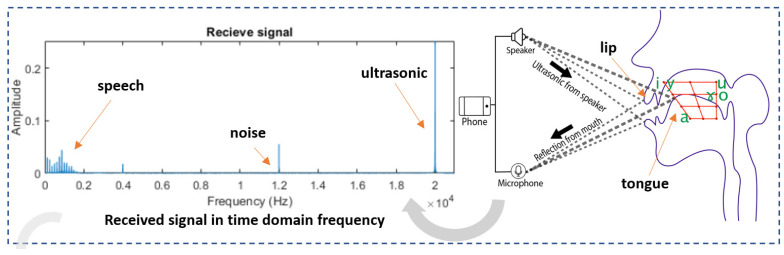
The sample signal in the frequency domain shows two ranges of acoustic signals: the speech signal and ultrasonic signal of the Doppler effect from the articulation movement.

We aim to employ the individual physiological and behavioral speech characteristics of vocal tract, tongue, and lip movements to provide a novel method for smartphone user authentication. We propose ArtiLock, a novel interaction system for authentication that can address the aforementioned shortcomings of speech technology and traditional articulation sensing methods based on smartphones by simultaneously using speech and ultrasonic signals to support user identification. A speech signal refers to an acoustic signal of a human voice below 5 kHz. An ultrasonic signal is an acoustic signal above 20 kHz that a human ear cannot receive. In addition, the available acoustic speaker and microphone are commonly assembled into a commercial smartphone that supports audio signals in the frequency range of up to 22 kHz or higher, covering both speech and ultrasonic signals. The key aspect of our approach is the use of a commercial smartphone to sense users’ speech organs based on pronunciation rules, by simultaneously analyzing speech and ultrasonic signals to sense articulation during utterances, as shown in Figure 1. We used monophthongs as the passphrase for the system because monophthongs are vowels that are the central component of a syllable described by phonemes. In addition, the phonemes of monophthongs are the smallest cluster of sounds within any language, and each vowel phoneme has unique articulation, giving it unique characteristics and qualities during articulation [8]. The tongue and lips are two articulators of the vocal tract that generate vowels. The proposed system employs speech signals to analyze articulation in the oral cavity, such as the tongue and vocal tract, and ultrasonic signals, such as the lip, in the external oral cavity.

This study focuses on articulation-based authentication and consists of six sections. It introduces the background of articulation-based applications and technology as well as the problems of existing articulation sensing technology. Section 2 discusses the current research status of mobile authentication approaches, and summarizes the challenges and corresponding solutions for articulation-based authentication. The proposed method for the articulation-based authentication experiment and data collection process is explained in Section 3. The results and discussion are presented in Section 4, and the conclusions are presented in Section 5. The contributions of this study are as follows.

- We propose a system that requires single utterances with respect to physiological and behavioral speech characteristics for smartphone user authentication, resulting in less calculations and resistance to attacks and environmental noise.
- We extract articulation features by simultaneously detecting audible (speech) and inaudible (ultrasonic) signals to build a user identification model based on individual articulation characteristics.
- We implement a prototype and verify the performance of the proposed system under real scenarios. The system is robust in resisting mimicry attacks in three environmental interferences with 99% accuracy, requiring few training samples.

## 2. Literature Reviews

### 2.1. Current Authentication Approaches of Mobile Devices

There are three main types of similar authentication schemes: graphical-alphabetic password-based authentication, physiological trait-based authentication, and behavior-based authentication. Typically, if the user does not use a smartphone for a long time, the phone automatically locks the screen, and the user needs to enter a password to access the phone’s main interface when using it again [9]. The unlocking scheme is widely used in different devices or applications but has apparent drawbacks. First, in public places, such passwords can be quickly and maliciously snooped. Second, an attacker can extract the fingerprint traces left on the screen and combine them with other personal information to infer the content of the password relatively easily. A previous study showed that most users have similar patterns when setting their passwords [10]. If others steal this information through specific technical means, an attacker is likely to obtain the correct password. User behavioral traits are similar to invisible passwords, unlike graphical-alphabetic passwords or fingerprints that can be compromised or stolen. However, a person’s behavioral traits are difficult to steal, and it is almost impossible to imitate an individual’s behavioral traits. Here, we introduce several existing authentication methods based on user behavioral habits. The first type is gesture-based authentication, in which a user must enter a predefined gesture on the screen when unlocking a device [11]. For example, Shahzad et al. proposed extracting the speed, magnitude, and duration of people’s gestures instead of gesture patterns as identity IDs; the error rate of this system was as low as 0.5% [9]. The second type of authentication is based on gait recognition, which extracts the differences in the gait of people walking for authentication [12]. However, gait recognition requires an extensive training sample set, and there are significant challenges in constructing gait models for users in different planes.

**Figure 2:**
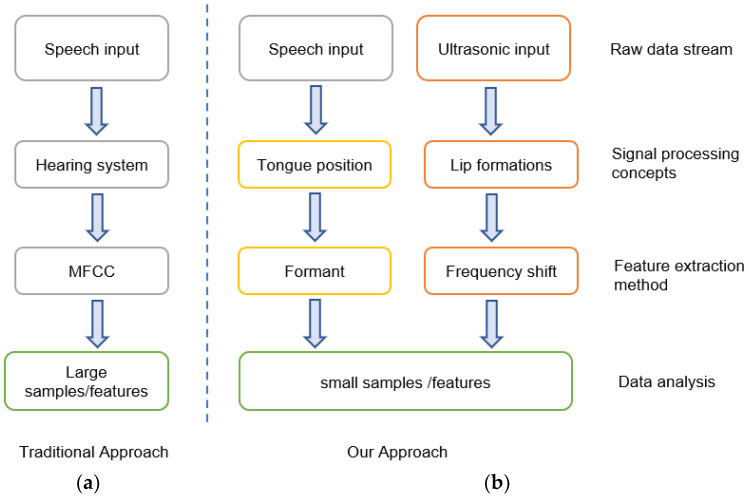
The system frameworks: (**a**) the traditional approach using the speech processing method converts the speech signal to a sequence of MFCCs, resulting in a large sample size; (**b**) the proposed system simultaneously uses speech and ultrasonic signals without requiring large sample sizes to support articulation-based identification.

Mel-frequency cepstral coefficients (MFCCs) are widely used to extract features for voice-based authentication [13,14,15,16,17,18]. MFCCs are obtained by extracting features from the audio signal, and when used as input to the base model, they produce much better performance than when directly considering raw audio signals as input. As shown in Figure 2, the typical speech processing method converts the speech signal from approximately 100 Hz to 5500 Hz into a sequence of MFCCs, and all features are then analyzed using a model such as a neural network or GMM [14,15,16,19]. However, excessive multiplication and the calculation process of MFCCs lead to high-capacity requirements of the system. The MFCC concept relies on a near approximation of the human hearing system [17]. The trade-off is that MFCCs are not robust against noise because of their dependence on the spectral form [13,17], which is not suitable for this scenario because it requires mobility in different scenarios and uses a smartphone for user verification based on sensing articulation, that is, the tongue and lip.

### 2.2. Articulation-Based Authentication and Current Limitations

Although voice authentication is generally secure, an attacker can easily trick it by using a prerecorded voice sample or mimic speaking habits. More secure authentication techniques based on automatic speech recognition or ASR have been proposed to overcome the shortcomings of traditional voice authentication methods, such as physiological and behavioral speech characteristics. (1) Physiological speech characteristics: people’s inherent physiological characteristics, such as fingerprints, facial features, and voice are used. Some liveness-based solutions have also been proposed. For example, VocalLock explored individual vocal tract uniqueness without specific passphrase content to realize voice-based user authentication [20]. VoiceLive finds live users by analyzing their unique speech systems and stereo recordings from their smartphones [21]. Because of this one-of-a-kind TDoA dynamic, the phone can detect the liveness close to the user’s mouth. These individual characteristics usually do not change over time and are unique; however, the data collection process is sensitive to environmental changes. Xu et al. proposed WaveEar, an end-to-end noise-resistant speech-sensing system that directs mmWave signals towards the near-throat region of the speaker to sense vocal vibrations [22]. However, their custom device is not well suited for ubiquitous use in mobile computing. Lippass and SilentKey recognized lip patterns using mobile phone ultrasonic signals [23,24]. However, they only recognize long passphrases in the vocabulary pattern because the training data requires a large amount of speech data in a practical scenario. Consequently, previous approaches relied on multiple syllables to produce unique features. The two methods cited above still require more than one syllable for authentication passphrases. This study presented two major challenges. First, the registration phase required a large sample size. Second, it is sensitive to the dynamic nature of the environment, for example, noise in the environment affects it.

## 3. Materials and Methods

### 3.1. Data Collection

**Figure 3:**
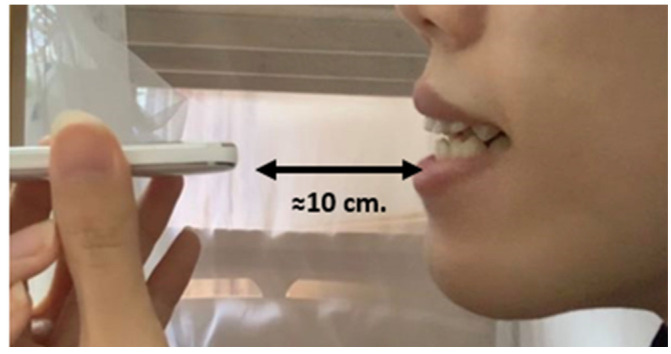
Setup of speaking position.

A monophthong is a simple vowel that is composed of a single utterance. Vowels are sounds that do not obstruct the airstream; specifically, the tongue and lips are the only articulators of the vocal tract that produce vowels [25]. This study aimed to examine whether the system can detect illegal users or resist attacks through articulation. Thus, the proposed system initially focused on uttering the six monophthongs in Mandarin [26]: /a/, /i/, /ɤ/, /o/, /u/, and /y/, to perform user identification through the articulation of individual physiology and behavior. The experiment involved 20 native Mandarin Chinese speakers aged 21 to 25 who were informed of the purpose of the experiment. We considered three different scenarios surrounding interference: in an office with conversations and HVAC (≈20 dB), in a noisy room (~50 dB), and outdoors (≈30 dB), as illustrated in the speaking position in Figure 3. We implemented a custom-made application that generated and recorded ultrasonic signals in Huawei Mate 9 (Shenzhen, China) for data collection.

In a mimicry attack, an attacker observes the user uttering a phrase and attempts to replicate the action, whereas a legitimate user sets passphrase during the registration process. We recorded the articulatory gestures of the participants as they spoke the passphrase, using a digital video recorder (GoPro, San Mateo, CA, USA). To preserve privacy, the video was recorded only in the lower facial region, which included the articulator movement of the lips and jaw. A total of ten attackers took part in the experiment, each mimicking one of the participants, were invited to carefully watch the video and repeatedly mimic the articulative gestures to learn how to imitate a legitimate user’s pronunciation. They were instructed to imitate the articulator’s speed, voice, and lip movements. When the attackers claimed that they had learned how the targeted user (a legitimate user) in the video spoke and moved the articulators, they began conducting mimicry attacks using the same smartphone used for authentication. Each participant was subjected to five trials for each of the six vowels, which resulted in 300 mimicry attacks.

An attack that eavesdrops on a legitimate user’s recorded voice is known as a playback attack. A particular form of each speaker replayed a recording from ten participants. To play back each pre-recorded voice sample, we utilized a BOSE Soundlink Mini II speaker (Hessen, Germany). Static playback attacks were conducted using stationary speakers approximately 10 cm from a smartphone. The same smartphone captured replay attacks in the same position as the participants used for authentication. The speaker contributed to the same five trials for each of the six vowels, resulting in 300 replay attacks.

### 3.2. System Overview

**Figure 4:**
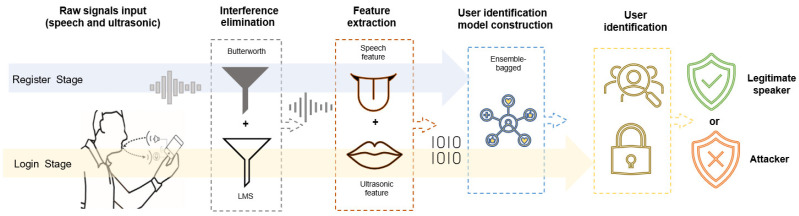
System framework of ArtiLock: (1) The interference elimination module removes the noise. (2) The speech feature extraction module extracts the oral articulation. (3) The ultrasonic feature extraction module envelopes mouth-reflected signals. (4) Authentication is performed by the user identification module using the training model.

The proposed system comprises four core modules, as shown in Figure 4. (1) The interference elimination module removes environmental signals such as noise, reflections from other objects, and signal disturbances introduced by hardware distortion. (2) The speech feature extraction module calculates the tongue position, vocal tract size, and pitch of speech signals. (3) The ultrasonic feature extraction module envelopes mouth-reflected signals. (4) The user identification module performs authentication based on the user identification model trained in the training phase.

#### 3.2.1. Register Stage

After the legitimate user “pronounces” the monophthongs on the device to provide training samples, the interfering signals are filtered for each training sample. In a near-field scenario, the articulation movement of the user causes speech and reflects the direction of the ultrasonic signal. Therefore, feature parameters are collected from these signals, which characterize the content of tongue position, lip motion, and vocal tract. Next, a user identification model was trained based on the extracted feature vectors to obtain an effective user identification model.

#### 3.2.2. Login Stage

The users “pronounce” the monophthong as a password to log in to the device, and the system filters and extracts the signal samples provided by the user and then employs the user identification model trained in the registration phase to classify and judge the test samples for user login authentication.

### 3.3. Interference Elimination

For the first type of interference, the daily communication voice signal frequency is 200–700 Hz, which is much lower than the 20 kHz used in this system and can be removed by filtering the signal at lower frequencies. Regarding the second type of interference, the speed of other body parts, such as the arm during movement, is usually between 0.25 m/s and 4 m/s [27]; this introduces a frequency shift to mouth movements of around 40 Hz. The frequency domain can be observed in the frequency patterns because the two interferences are more significant than the frequency introduced by mouth movements. Thus, a Butterworth bandpass filter was used to solve the first type of interference. This system sets the passband range from 19,960 to 20,040 Hz to eliminate external environmental noise. The transfer function coefficients of the filter are returned as row vectors of length 2*n* + 1 for a bandpass filter, which are expressed in terms of *b* and *a* as (1) for digital filters. The filter order consists of the numerator and denominator coefficients, and *X*(*z*) and *H*(*z*) represent the input and output signals, respectively. In bandpass designs, *n* represents half of the filter order.

(1) $$H\left(z\right)=\frac{B\left(z\right)}{A\left(z\right)}=\frac{b\left(1\right)+b\left(2\right)z^{-1}+...+b\left(n+1\right)z^{-n}}{a\left(1\right)+a\left(2\right)z^{-1}+...\;+a\left(n+1\right)z^{-n}}X\left(z\right)$$

**Figure 5:**
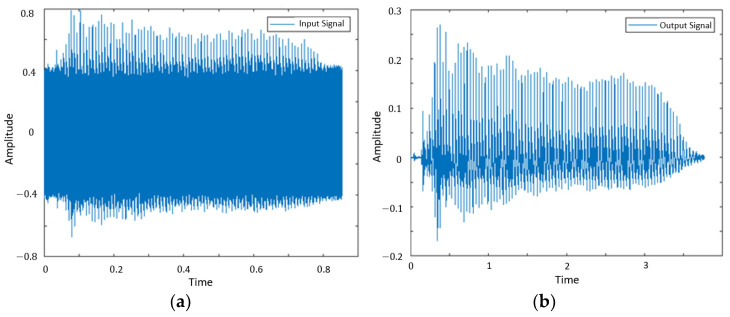
Sample signal showing both speech and ultrasonic signals in the time domain: (**a**) received signal and (**b**) filtered signal.

**Figure 6:**
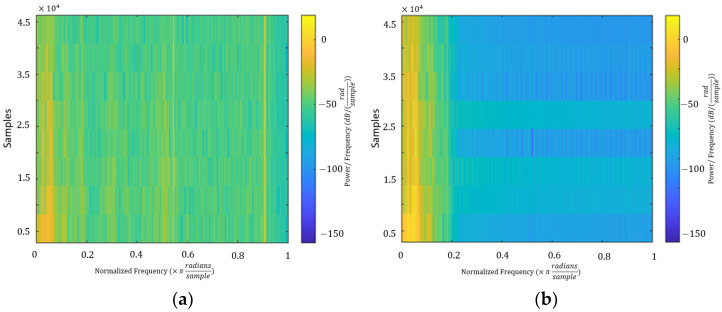
Spectrograms of sample signals: (**a**) received signal and (**b**) filtered signal.

Because such body movements or handshaking interferences are not natural to predict in advance, adaptive filters are suitable for this case. The least mean squares (LMS) algorithm was used here because it requires fewer hardware resources and has good traceability [28]. The step size of the LMS is fixed at 0.5, to balance the errors and fast convergences [29]. The effect of interference elimination after applying the Butterworth bandpass filter and LMS filter in the time-domain frequency is shown in Figure 5, and the spectrograms are shown in Figure 6.

### 3.4. Extract Feature Articulator from Ultrasonic Signals

When relative motion occurs between the two sources and receivers, the Doppler effect occurs and variation occurs in the frequency of a wave in response to the motion of an observer relative to the wave source. The mathematical expression (2) for the Doppler effect is as follows:

(2) $$f^{\prime }=\left(\frac{c\pm v_{r}}{c\pm v_{t}}\right)f_{0}$$

As the speaker moves toward the receiver at a constant speed *v*, the signal’s frequency received by the receiver changes. When the sound source or speaker of the waves moves toward the receiver, the arrival time of each sound wave is shorter than the previous one. Thus, the distance between the peaks decreases, that is, the wavelength of the signal decreases, which means that the receivers perceive an increase in sound wave frequency. Conversely, as the source moves farther away from the receivers, the wavelength of the receivers increases, thereby decreasing the frequency. We analyzed the signals via their frequency domain, each corresponding to lip motion. Our objective was to establish a connection between the frequency envelope and the lip shape of each monophthong. The Doppler frequency envelope from the ultrasonic signal generated at 20 kHz obtained by comparing the lip movement peaks is approximately 40 Hz or from 19,960 Hz to 20,040 Hz with a central peak at 20 kHz.

**Figure 7:**
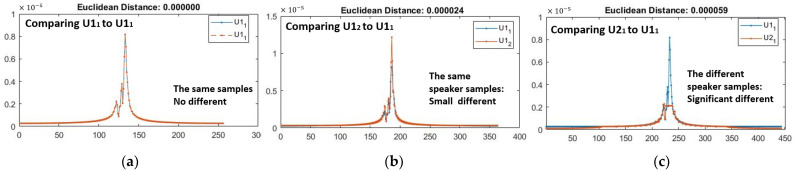
A comparison of the three enveloped reflected signals in the frequency domain is made when U1 and U2 pronounce the vowel “a”: (**a**) Comparing the same sample of U1; (**b**) Comparing two samples of the same passphrase of U1; (**c**) Comparing the same passphrase of U1 and U2.

**Figure 8:**
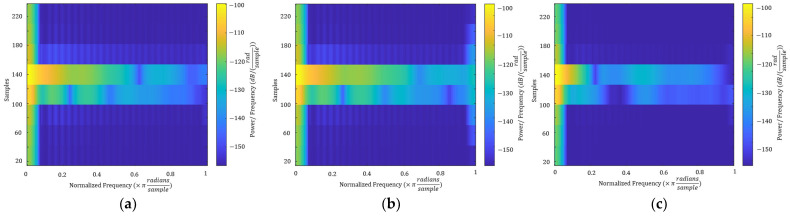
A comparison of the three enveloped reflected signals in the spectrogram is made when U1 and U2 pronounce the vowel “a”: (**a**) Comparing the same sample of U1; (**b**) Comparing two samples of the same passphrase of U1; (**c**) Comparing the same passphrase of U1 and U2.

According to the signal interference principle, when users utter the same or different sounds, a difference occurs in the mouth movement owing to the users’ physiology and habits; thus, the interference phenomenon causes the reflected signal to have a different envelope shape. The findings of this study show that signals from an exact speaker yield minor differences in lip shape, based on Euclidean distances. Figure 7 demonstrates the envelope changes of the reflected signals when two users (U1 and U2) pronounce the vowel “a” as the same password. The shapes of the envelopes from the same user are very similar, whereas the envelopes of the two different users differ significantly. Measuring the distance between two signal envelopes can differentiate users, but it may require more calculations and feature vectors. Based on the findings of this study, it is evident that signals from the exact speaker yield minor differences in Euclidean distances. Therefore, we extracted the mean frequency, power, bandwidth, and medium from the enveloped ultrasonic signals. Figure 8 shows the spectrograms of the same three reflected signals of two users (U1 and U2), as shown in Figure 6. The spectrograms also revealed that individuals produced the same lip movement, resulting in the same signal pattern in the bandwidth.

After eliminating the interference, we extracted five features from the frequency domain to analyze the lip movement patterns using Algorithm 1. The envelopes of the reflected signals were extracted using the Hilbert transform. The Hilbert transform was applied to obtain the envelope of the signal and construct the trajectory of the mouth movement [23]. The Hilbert transform was calculated using (3). The Hilbert envelope spectrum can be expressed as

(3) $$h\left(f\right)=\int_{-\infty }^{\infty }\sqrt{x^{2}\left(t\right)+{\hat{x}}^{2}\left(t\right)}\exp\left(-j2\pi ft\right)dt$$

where *x*(*t*) is the given signal. The envelope was downsampled and normalized to 256 to reduce computational effort using the pwelch function. The downsampled envelope signals are then estimated as the occupied bandwidth, median frequency, and mean frequency of the signal, and the power bandwidth contained in an ultrasonic frequency band. The bandwidth that contains 99% of the total integrated power of the transmitted spectrum, centered on the allocated channel frequency, is referred to as the occupied bandwidth. The occupied bandwidth algorithm estimates the periodogram power spectral density using a rectangular window, and then integrates it using the midpoint rule. The occupied bandwidth is the frequency difference between the spots in the spectrum where the integrated power exceeds 0.5% and 99.5% of the total power [30]. The power bandwidth estimates the periodogram power spectrum using a rectangular window, and uses the maximum estimate as the reference level [31]. Bandwidth is defined as the frequency difference between two places on the spectrum that is at least 3 dB below the reference level. If the signal reaches one of its endpoints before decreasing by 3 dB, the power bandwidth computes the difference by using the endpoint. Bandwidth is defined as the frequency difference between two locations on the spectrum that is at least 3 dB below the reference level. If the signal reaches one of its endpoints before decreasing by 3 dB, the power bandwidth computes the difference by using the endpoint. The mean and median frequencies of the power spectrum are widely used as indicators of spectral changes. In various studies, the mean frequency is referred to as the mean power frequency or the mean spectral frequency. The mean frequency of a spectrum is computed as the product of the spectrogram intensity and the frequency divided by the total spectrogram intensity. Random noise has a minor effect on the estimation of the median frequency, particularly noise in the high-frequency band of the power spectrum. The median frequency was determined when the power spectrum was divided into two sections of equal amplitudes. The median frequency is defined as half of the total power, or the area of the total power divided by half.
**Algorithm 1** Ultrasonic Signals Articulation Feature Extraction Algorithm**Input:***in_sig*: the input signals; **Output:***env_mean*: the mean of two characteristics; *env_meanfreq*: the mean normalized frequency of the two characteristics; *env_bw*: the power bandwidth of the two characteristics; *env_obw*: the occupied bandwidth of the two characteristics; *env_medf req*: the median normalized frequency of the two characteristics; 1: *bw_sig*[*i*] *← filterButterworth*(*in_sig i*]) 2: *lms_sig*[*i*] *← filterLMS*(*bw_sig* [*i*]) 3: **for**
*i ←* 0 *to* 1 **do** 4:   *h ← hibertFun*(*in_sig* [*i*]) 5:   *p ← pwelchFun*(*in_sig* [*i*]*, h*) 6:   *e ← envelopeFun*(*p*) 7:   *env*[*i*] *← e* 8: **end for** 9: *env ← dwtFun*(*env* [0]*, env* [1]) 10: *env_meanfreq ← meanFreq*(*env*) 11: *env_bw ← powerbw*(*env*) 12: *env_obw ← obw*(*env*) 13: *env_medfreq ← medFreq(env)*

### 3.5. Extract Feature Articulator from Speech Signals

This system adopts linear predictive coding for speech feature extraction (formant, pitch, and vocal tract) because it imitates human vocal tract function [17]. First, we need to set a preprocessing preset. The effective duration of the analysis window is measured in seconds, and a double length exists that employs a Gaussian-like analysis window with sidelobes below −120 dB. We selected a window length of 0.025 s, and the actual duration of the Gaussian window was 0.050 s [32]. After setting the pre-emphasis and windowing, we extracted the formant frequency by computing the linear predictive coefficients using the Burg algorithm [33]. The formant frequencies are referred to as F1, F2, F3, and F4, from low to high. The formant or frequency of their resonance peaks differentiates vowels; for example, F1 represents tongue height, whereas F2 represents tongue-backness, as depicted in Figure 1 and Table 1, which are mapped to the vowel diagram to estimate the position of the tongue.

The fundamental concept of linear prediction is the correlation between the speech sample values. The current speech sample value can be approximated by combining several past-speech sample values. A unique set of prediction coefficients is determined by minimizing the square sum of the difference between the actual speech sample value and predicted value [34]. The LPC algorithm is mainly divided into the establishment and solution of linear prediction. Specifically, the inputs of the algorithm were audio data, linear prediction order, and sampling frequency. First, the autocorrelation function is calculated for each order, and then the prediction coefficient of the *i* + 1 order is recursively solved using the *i*th order prediction coefficient. The first step of the recursive algorithm is to initialize the prediction coefficients and errors of the 0th order and the 1st order. It is then solved through a loop according to Equation (4), given the coefficients of the *k*th order.

(4) $$k_{i}=\frac{\lambda^{\left(i-1\right)}}{E^{\left(i-1\right)}}=\frac{R\left(i\right)-\sum_{k=1}^{i-1}a_{k}^{\left(i-1\right)}R\left(i-k\right)}{E^{\left(i-1\right)}}$$

The *λ* value of the *k*th order was obtained according to Equation (5) to obtain the *k* + 1st-order prediction coefficient.

(5) $$Fa_{j}^{\left(i\right)}=a_{j}^{\left(i-1\right)}-k_{i}a_{i-j}^{\left(i-1\right)},\;a_{i}^{\left(i\right)}=k_{i}$$

After obtaining the prediction coefficient *a_i_*, the polynomial coefficient of the linear prediction filter *A*(*z*) must be decomposed, and its complex polynomial root must be obtained. The *a*tan^2^ form was used to obtain *θ* for each complex root. Finally, the formant can be calculated using Equation (6), according to the frequency *f_i_* and bandwidth *B_i_*. The array elements are then returned to obtain the formant frequency.

(6) $$f_{i}=\frac{\theta_{i}}{2\pi T},\;B_{i}=\frac{-lnr_{i}}{\pi T}$$

**Figure 9:**
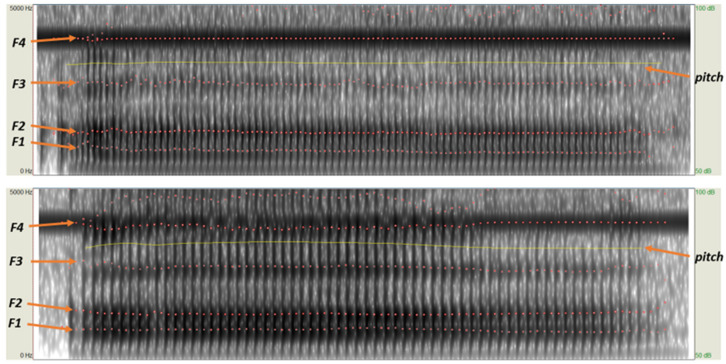
An example of a comparison in the time-domain of the formant frequency, pitch, and vocal tract is made when User 1 and User 2 pronounce the vowel “a”. Based on the findings of this study, it is evident that signals from the exact speaker yield minor differences in the tongue and vocal tract.

Another considerable variation exists between speakers: the vocal tract size difference between a person’s front and back vowels might be more significant than the average vocal tract length difference between men and women [35]. After obtaining the formants, vocal tract length estimation in the neutral configuration (the position of the vocal organs where a tube without obstacles is created from the larynx to the lips) must be performed. Equation (7) was applied to calculate the average frequencies of a tube closed at one end for vocal tract length extraction [25]:

(7) $$Fn=\frac{\left(2n-1\right)}{4L}$$

where *n* is the formant, *L* is the tube length, and *c* is the sound speed. Because we apply LPC, we can then use autocorrelation to estimate the pitch, which is acoustic periodicity detection based on the autocorrelation method that is more accurate, noise resistant, and more robust than the cepstrum or the original autocorrelation method [36]. Figure 9 visualizes the formant frequency, pitch, and vocal tract in the time domain of User 1 and User 2 pronouncing vowel “a”. We extracted features from speech signal using Algorithm 2.
**Algorithm 2** Speech Signals Articulation Feature Extraction Algorithm**Input:** *data*: audio data; *order*: order; *fs*: sampling frequencies; **Output:** *fre*: formant frequencies; *pre_pitch*: pitch estimation of data; *vol_tlenh*: volcal track length of data; 1: r: autocorrelation coefficient; a: prediction coefficient; 2: e: mean square error; N: length of data; 3:  **for***i* ← 0 *to order*
**do** 4:   **for**
*i* ← 0 *to N − i*
**do** 5:  *r*[*i*] ← *r*[*i*] + *data*[*n*] ∗ *data*[*n* + *i*] 6:  **end for** 7: **end for** 8: Initialize a[0],a[1],e[0] and e[1]. 9: **for**
*k* ← 1 *to order*
**do** 10:  **for**
*j* ← 0 *to k* + 1 **do** 11:  *λ* ← *λ − a*[*j*] ∗ *r*[*k* + 1 − *j*] 12:  **end for** 13:   *λ* ← *λ* ÷ *e*[*k*] 14:  **for**
*i* ← 1 *to k* + 1 **do** 15:  *U* [*i*] ← *a*[*i*] 16:    *V* [*k +* 1 − *i*] ←*a*[*i*] 17:  **end for** 18:   **for**
*i* ← 0 *to k* + 2 **do** 19:  *a*[*i*] ← *U* [*i*] + *λ ∗ V* [*i*] 20:  **end for** 21:  *e*[*k* + 1] ← *e*[*k*] ∗ (1 − *λ*^2^) 22: **end for** 23:  *rts* ← *findRoots*(*a*) 24:  **for**
*i* ← 0 *to rts.length*
**do** 25:     *arg* ← *atan*2(*rts*[*i*]*.imaginary, rts*[*i*]*.real*) 26:      *argList.add*(*arg*) 27: **end for** 28: *argSort*(*argList*) 29:  **for**
*i* ← 0 *to rts.length*
**do** 30:  *freqs*[*i*] ← *argList*[*i*] ∗ (*fs/*2*π*) 31: **end for** 32: *pre_pitch* ← *esPitch*(*data*) 33: *vol_tlen* ← *volcalTrack*(*data*) 34:  return *pre_pitch*, *vol_tlen*, *freqs*;

### 3.6. User Identification Model

#### 3.6.1. Classification Model

An ensemble classifier is suitable as a user identification model because a weighted combination of multiple classification models increases the predictive performance. Bagged tree kernels are the first reasonable choice for this model because a decision tree exhibits unparalleled performance by weighting the results of the tree, reducing the variance, and overfitting the dataset. A bagging integrated classifier combines multiple “weak” decision trees into one “strong” decision tree with high efficiency and accuracy. There are 10 input features: F1, F2, F3, F4, vocal tract, pitch, frequency mean, power bandwidth, occupied bandwidth, and frequency medium. In addition, a grid search algorithm was applied to optimize the appropriate hyperparameters. We set the search range to be ten as the default with 5-fold validation. The model was trained using the learning classification toolbox on MatLabR21a on a PC with an Intel i7 CPU (Santa Clara, CA USA), 16GB RAM, and 1TB SATA.

#### 3.6.2. Evaluation Metrics

The following metrics were used to evaluate the proposed system. The false acceptance rates or FAR scales for attackers that are accepted as legitimate users. The FAR can be calculated using Equation (8):

(8) $$\mathrm{FAR}=\frac{\mathrm{Number}\;\mathrm{of}\;\mathrm{attakers}\;\mathrm{samples}\;\mathrm{accepted}\;\mathrm{as}\;\mathrm{legitimated}\;\mathrm{user}}{\mathrm{Total}\;\mathrm{number}\;\mathrm{of}\;\mathrm{legitimated}\;\mathrm{user}\;\mathrm{samples}}\;\times \;100\%$$

The false rejection rate (FRR) is computed as the probability of faults identifying a legitimate user. The FRR equation is given by Equation (9):

(9) $$\mathrm{FRR}=\frac{\mathrm{Number}\;\mathrm{of}\;\mathrm{legitimated}\;\mathrm{user}\;\mathrm{samples}\;\mathrm{rejected}}{\mathrm{Total}\;\mathrm{number}\;\mathrm{of}\;\mathrm{legitimated}\;\mathrm{usersamples}}\;\times \;100\%$$

An equal error rate (EER) is the rate at which the false acceptance rate equals the false rejection rate. A lower EER yields a better performance. The EER equation is given by Equation (10):

(10) EER=false acceptance rate = false rejection rate

The receiver operating characteristic (ROC) curve describes the relationship between the true and false acceptance rates while varying the detection threshold. Furthermore, the identification accuracy rate (ACC) indicates the possibility that the system accepts legitimate users and rejects others. It is then given as Equation (11).

(11) $$\mathrm{ACC}=\frac{\mathrm{true}\mathrm{acceptance}\mathrm{rate}+\mathrm{true}\mathrm{rejection}\mathrm{rate}}{\mathrm{Total}\mathrm{number}\mathrm{of}\mathrm{samples}}\;\times \;100\%$$

## 4. Results and Discussion

### 4.1. Performance on User Identification

**Figure 10:**
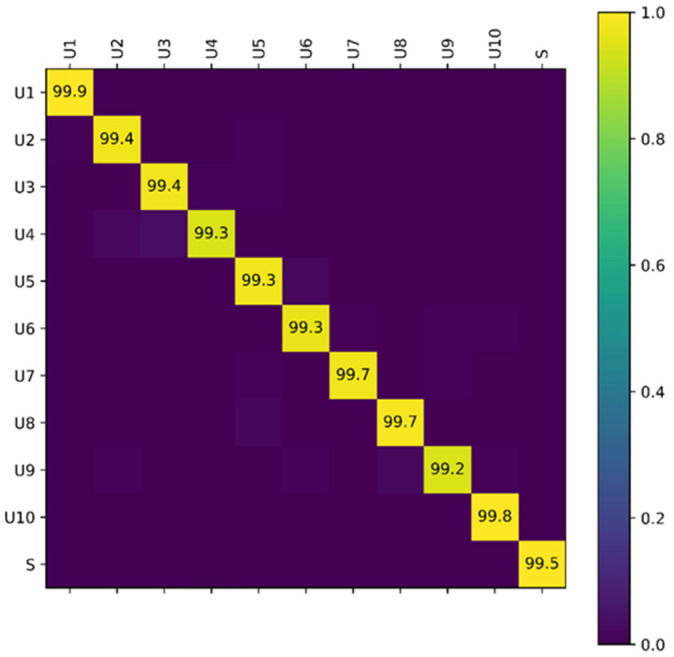
Confusion matrix of ArtiLock in the register stages.

We present a novel voice-based user authentication approach that employs individual physiological speech characteristics and behavioral speech characteristics by sensing the articulation of single utterances. First, we evaluated the overall performance of ArtiLock for user identification. Figure 10 shows the confusion matrix of ArtiLock, each entry of which is the average accuracy of the three different environments. ArtiLock can achieve an average accuracy of 95.51% in terms of identifying legitimate users and an average accuracy of 99.51% for detecting non-legitimate users. Overall, the average accuracy of ArtiLock user identification was 99.48%.

### 4.2. Performance of Attack Resistance

**Figure 11:**
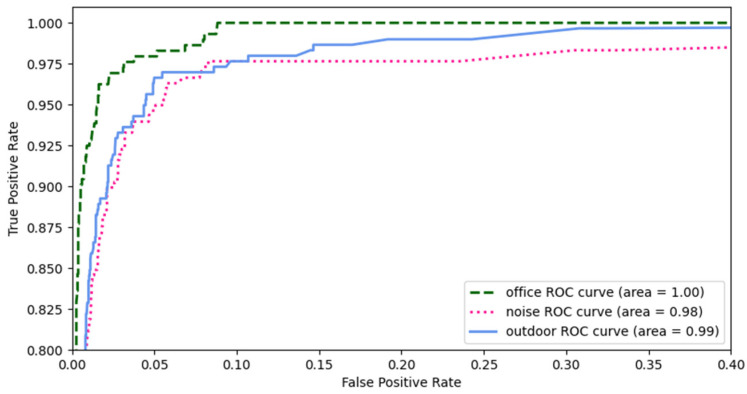
ROC curves of our system under attack.

This study presents a new approach for user identification that employs individual physiological and behavioral speech characteristics by sensing the articulation of single utterances. First, we present the overall system performance in the three environments under playback and mimicry attacks. Figure 11 shows the overall system performance based on the receiver operating characteristic or ROC of our system under both types of attacks. The proposed system reveals false acceptance rates of 2.17%, 5.85%, and 2.25% under office, noisy, and outdoor conditions, respectively, whereas the true acceptance rates are as high as 97.70%, 94.15%, and 95.54%, respectively. ROC analysis indicates that the combination of physiological speech and behavior characteristics can effectively detect legitimate users in various environments under playback and mimicry attacks.

### 4.3. Performance in Different Environments

**Figure 12:**
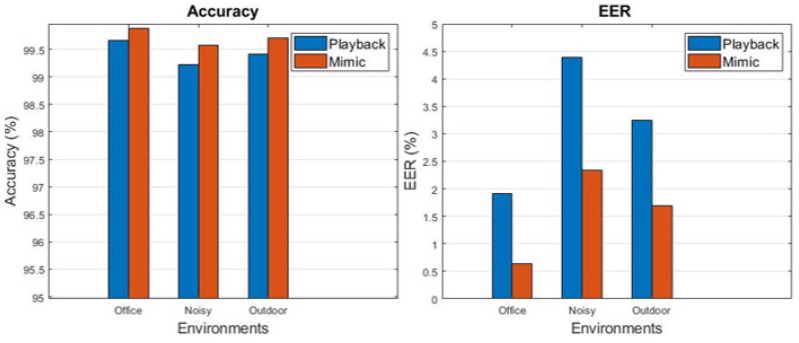
Accuracy and equal error rates for the three scenarios.

Figure 12 summarizes the accuracies and equal error rates under replay and mimic attacks in three different environments. In all cases, the accuracies were greater than 99%, and the errors were less than 4%. The average accuracy is 99.66% in offices, 99.40% in noisy environments, and 99.77% outdoors. The equal error rate or EER indicates the rate at which the false acceptance rate equals the false rejection rate, averaging 1.28% in the office, increasing to 3.37% when the environment is noisy and 2.48% when outdoors. As a result of this study, the extracted features from the speech and ultrasonic signals of vowel phoneme sounds could be applied to capture the differences in articulation between legitimate users and attackers in various interference conditions. The robustness of ArtiLock can be attributed to two key factors: (1) the frequency of speech is lower than that of an ultrasonic signal, which is easily filtered; and (2) the surrounding movements influence a lower frequency than lip movements, which the system can distinguish. Therefore, the proposed scheme is resistant to environmental interference.

### 4.4. Impact of Training Data Size

**Figure 13:**
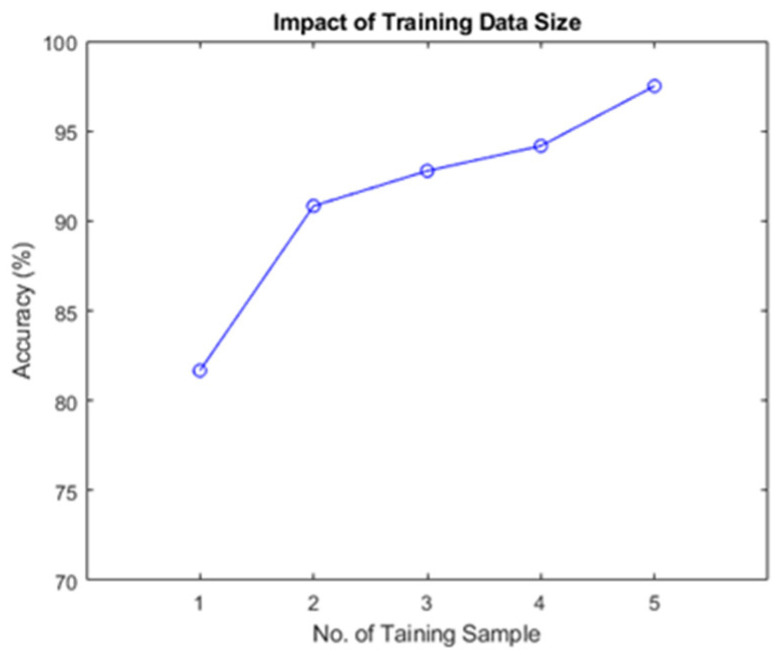
Accuracy of different numbers of samples in the registration stage.

The training data size was the number of speaking times during the registration stage. Authentication accuracy and user experience are affected by the number of training samples. A more significant number of training samples will result in a more robust system accuracy rate; however, the cumbersome and lengthy acquisition process in the registration phase will result in a poor user experience. Therefore, this experiment aims to determine the appropriate number of training samples by evaluating the impact of the sample size on system performance. Figure 13 shows the accuracy of ArtiLock for different sizes of training data or registration stages. The results also indicate that accuracy increases as the number of training data samples increases. The accuracy first increased significantly from one to two samples and then steadily increased from two to five samples. ArtiLock achieves an accuracy of approximately 98% when a user speaks five times in the register stage. More speaking time does not contribute to an improvement in ArtiLock accuracy. Even when speaking three times during the register stage, ArtiLock achieved an accuracy of 93%. ArtiLock constructs a user-identification model based on articulation sensing, which requires users to provide only a few training data samples. Existing voiceprint-based user authentication usually requires a user to speak a non-monosyllable passphrase; however, it also requires a long passphrase length. For example, WeChat requests that users read at least eight digits during the register stage [3]. iOS requires users to say “Hey, Siri” to set up voice recognition [37]. Despite this, our proposed system requires only a single utterance; ArtiLock can achieve a user-friendly experience while maintaining a superior user authentication performance.

### 4.5. Comparison with Other Authentication Systems

The proposed approach was compared with state-of-the-art approaches developed using the same settings of a single utterance: LipPass [23], SilentKey [24], and VocalLock [20]. The articulation features, approach, and performance of the state-of-the-art and proposed approaches to articulation-based schemes have similar accuracy rates, as shown in Table 2. Our experimental results revealed that our proposed system produces better results than all state-of-the-art approaches, achieves single-syllable passphrases, and requires fewer training samples to resist surrounding interference. Because ArtiLock employs speech and ultrasonic signals toward individual physiological and behavioral speech characteristics, the system ubiquitously obtains satisfactory accuracy through a smartphone, without requiring a specific tool.

### 4.6. System Advantages Ages

As a result of authentication methods based on physiological characteristics, others cannot copy or reproduce the articulation password. Compared with other authentication methods based on behavioral traits, this system has a higher recognition accuracy rate and is more adaptable to environmental variability. It is also easier to use than the gait or fingerprint data-collection process. The system can compensate for this deficiency, and the user only needs to utter a single monophthong syllable to perform identity authentication. The system only requires users to provide five training samples in the registration stage to improve user experience. The advantages of this system are as follows:

- Strong attack resistance: The proposed system is an authentication technology that integrates physical and behavioral characteristics of how people speak with different speech organs and articulation movements and is robust to mimicry attacks in three environmental interferences with 99% accuracy. Compared with traditional PINs, fingerprints, and other passwords, using articulation as passwords cannot be fully replicated by any hardware device or other means. Even if an attacker learns the content of a password, he or she cannot reproduce the user’s articulation or movement pattern, and thus, malicious attacks can be avoided.
- High usability: Compared with other behavioral habit-based authentication techniques that require a large amount of training data, such as gait-based schemes that require 40 training samples from the user [38], our system requires less than five training samples to achieve the same level of correctness. Therefore, the system considers the shortest possible training process to provide a better user experience while ensuring reliability.
- User friendly: We simultaneously detect audible (speech) and inaudible (ultrasonic) signals to extract articulation features from single utterances based on physiological and behavioral speech characteristics for smartphone user authentication. Registration for the system is easy because it only requires the user to create a small number of samples in a single syllable. In addition, the acoustic system is resistant to light sensitivity in a dim environment compared with image-based authentication, which requires sufficient light, for example, Apple’s Face ID [7].

### 4.7. Limitations

ArtiLock is a research prototype with strict limitations and is not a commercial product. First, ArtiLock was evaluated by subjects with limited ages and education levels. It will be helpful to consider a system with a more significant number of participants with a more diverse background to better understand performance. Moreover, the system was evaluated for only a few months. A long-term study could be conducted considering that these individual characteristics are likely to change over a lifetime, such as changes in mouth cavities or a user growing a beard. ArtiLock can serve as an auxiliary channel for two-factor authentication. ArtiLock explores the static shape and dynamic movements of the lip, tongue, and vocal tract as biometrics to realize user authentication, potentially serving as a primary authentication factor. Nevertheless, periodically updating user profiles can mitigate such limitations. Finally, the system requires users to hold their phones close to their mouths to capture the articulatory gesture reliably. This may limit the application of the system. For instance, the system is less applicable to cases where the phone is not held in the user’s hand but instead is placed somewhere in proximity.

## 5. Conclusions

ArtiLock is a smartphone-based articulator detection system that can accurately identify speakers without requiring users to perform cumbersome operations. The objective of the proposed system is to verify the speaker by examining the physiological and behavioral speech characteristics of the articulation movement while speaking, which reduces the number of samples in the registration stage and avoids complicated feature extraction. Our approach leverages speech signals and the Doppler effect of ultrasonic signals, which transmit a high-frequency acoustic signal from the built-in speaker and the microphone receives reflections. The system achieves high accuracy and a low error rate in the three environments, works well even with a single utterance, and requires few sample sets for training.

## Figures and Tables

**Table 1:** Monophthongs and articulations in Mandarin Chinese.

Tongue-Height	Tongue-Backness			
	Front		Back	
Lip shape	unrounded	rounded	unrounded	rounded
**Hight**	/i/	/y/		/u/
**Mid**			/ɤ/	/o/
**Low**	/a/			

**Table 2:** Comparison with other articulation-based authentication systems.

System	ArtiLock	LipPass	SilentKey	VocalLock
Performance	99.5% ACC	90.2% ACC	82.5% ACC	91% ACC
No. of required samples	1–5	3–10	5–9	1–5
Different Passphrase	6	10	6	20
Passphrase required length	1	More than 4	4–6	3–12
Frequency band	Speech and Ultrasonic	Ultrasonic	Speech	Speech

## Data Availability

Not applicable.
